# 儿童高级别B细胞淋巴瘤11例临床分析

**DOI:** 10.3760/cma.j.issn.0253-2727.2023.02.012

**Published:** 2023-02

**Authors:** 娟 刘, 娟 王, 佳 朱, 玉 张, 素英 路, 斐斐 孙, 俊廷 黄, 艳鹏 伍, 凤银 蔡, 瑞卿 蔡, 子俊 甄, 晓非 孙, 翼鷟 张

**Affiliations:** 1 中山大学肿瘤防治中心儿童肿瘤科，华南肿瘤学国家重点实验室，肿瘤医学协同创新中心，广州 510060 Department of Pediatric Oncology, State Key Laboratory of Oncology in South China, Collaborative Innovation Center for Cancer Medicine, Sun Yat-Sen University Cancer Center, Guangzhou 510060, China; 2 广州医科大学附属第五医院儿童肿瘤科，广州 510700 Department of Pediatric Oncology, The Fifth Affiliated Hospital, Guangzhou Medical University, Guangzhou 510700, China; 3 中山大学肿瘤防治中心病理科，华南肿瘤学国家重点实验室，肿瘤医学协同创新中心，广州 510060 Department of Pathology, State Key Laboratory of Oncology in South China, Collaborative Innovation Center for Cancer Medicine, Sun Yat-Sen University Cancer Center, Guangzhou 510060, China

高级别B细胞淋巴瘤（HGBL）是2016年WHO淋巴瘤分类中一类介于弥漫大B细胞淋巴瘤（DLBCL）和伯基特淋巴瘤（BL）之间的B细胞淋巴瘤，具有高度侵袭性。包括伴MYC和BCL-2和（或）BCL-6重排的淋巴瘤和不伴MYC和BCL-2或MYC和BCL-6基因合并易位的非特指型（NOS）淋巴瘤两种类型[1]–[2]。国内外报道均表明HGBL患者的预后较差[3]–[4]，且治疗方案尚不统一，而儿童及青少年群体中HGBL相关研究更少。本研究回顾性分析中山大学肿瘤防治中心收治的11例儿童及青少年HGBL患者的病例资料，探讨其临床特点、病理特征和生存情况。

## 病例与方法

1. 病例：本研究纳入中山大学肿瘤防治中心儿童肿瘤科2010年5月至2020年10月收治的病理诊断为HGBL、年龄≤18岁的初诊患者，病理切片调出后由≥2位高年资病理医师重新判读。入组标准：①患者治疗过程中有准确的病历资料及规律的随访；②病理学确诊为HGBL；③患者初次就诊时未接受外院的其他化疗。排除标准如下：①曾患其他恶性肿瘤；②因患者自身原因导致治疗不规范或失访。本研究通过了中山大学肿瘤防治中心伦理委员会（IRB）的批准（B2022-494-01）。

2. 诊断标准：HGBL的病理形态学及免疫组化是确诊的主要标准，目前根据2016版WHO病理标准诊断。HGBL的病理特征介于DLBCL和BL之间，肿瘤细胞核大，核仁明显，核分裂象易见，有时呈现中等大小或以淋巴母细胞样为主，细胞核呈圆形，染色质分散，细胞核不明显或呈BL样，有时镜下可见星空现象。根据2016版WHO标准，其免疫组织化学（IHC）显示所有细胞均表达CD20，大多数表达Bcl-6，而CD10、Ki-67、MYC的表达呈现多样性，大多数不表达IRF4及MUM1。因其组织形态学及免疫表型较为多样，病理诊断难度大，多数情况下需要排除其他B细胞淋巴瘤而确诊。HGBL可分两种类型：①HGBL伴MYC和BCL-2和（或）BCL-6重排，包括所有伴随MYC和BCL-2和（或）BCL-6重排的B细胞淋巴瘤（除外一些罕见的滤泡性淋巴瘤和B细胞淋巴母细胞白血病/淋巴瘤），也称为双打击或三打击淋巴瘤；②HGBL NOS，包括介于DLBCL和BL之间或表现为淋巴母细胞样形态，但不伴MYC基因和BCL-2基因或MYC基因和BCL-6基因的合并易位。

3. 分期及危险度分级：中山大学肿瘤防治中心儿童HGBL在2015年之前采用St. Jude分期系统，2015年开始采用2015年修订的国际儿童NHL分期系统（IPNHLSS）。根据临床分期、手术切除程度及乳酸脱氢酶（LDH）将其分为极低危组、低危组、中危组和高危组。

4. 治疗方案：在儿童肿瘤科就诊的初诊HGBL患者接受了改良NHL-BFM-90/95方案（包括地塞米松、甲氨蝶呤、依托泊苷、长春新碱、长春地辛、异环磷酰胺、阿糖胞苷、环磷酰胺、吡柔比星、利妥昔单抗）治疗[5]，在成人肿瘤内科治疗的初诊HGBL患者接受了R-CODOX-M/R-IVAC方案（利妥昔单抗、环磷酰胺、长春新碱、吡柔比星、甲氨蝶呤/利妥昔单抗、异环磷酰胺、依托泊苷、阿糖胞苷）等治疗[6]。

5. 随访：随访方式为门诊复查及电话随访。患者结束治疗后的第1年每3个月复查1次，第2年每半年复查1次，第3年及以后每年复查1次。末次随访日期为2021年10月29日。

6. 疗效评估：儿童HGBL疗效评估参考国际儿童非霍奇金淋巴瘤疗效评价标准。总生存（OS）期为患者开始治疗至死亡或末次随访时间，无事件生存（EFS）期为患者开始治疗至疾病进展、死亡、发生致死性或不能耐受的不良反应或第二肿瘤的时间。

7. 统计学处理：定量资料用中位数（范围）或均数±标准差表示，采用Kaplan-Meier法计算OS率、EFS率。

## 结果

1. 基线资料：共纳入11例患儿，其中男10例，女1例，中位年龄8.6（3.6～12.5）岁。3例患者年龄≤6岁，8例患者>6岁且≤18岁。11例患者均累及结外器官：病灶位于腹、盆腔者7例，位于头面部者4例。Ⅱ期6例，Ⅲ期2例，Ⅳ期3例。初诊时LDH≥1 000 U/L者1例，≥500 U/L且<1 000 U/L者6例，<500 U/L者4例。极低危组1例，低危组5例，中危组3例，高危组2例。

2. 临床表现：患者的临床表现通常与肿瘤的发生部位密切相关，7例病灶位于腹、盆腔的患者中有6例腹痛，2例肠套叠，1例腹泻伴便血，1例呕吐；4例患者病灶位于头面部，其中2例表现为口咽部包块，1例表现为颈部包块，1例为右耳垂下包块。

3. 免疫组化及分子检测结果：IHC：8例CD10^+^CD20^+^ Bcl-2^+^Bcl-6^+^，其中4例C-myc^+^；1例CD10^+^CD20^+^Bcl-2^+^；1例CD10^+^CD20^+^Bcl-6^+^C-myc^+^；1例CD20^+^Bcl-2^+^Bcl-6^+^C-myc^+^。11例患者Ki-67均大于75％，6例MUM1^+^，7例TdT ^−^。FISH：1例MYC^+^BCL-2^−^BCL-6^−^，6例MYC^−^BCL-2^−^BCL-6^−^，1例MYC^+^BCL-2^−^（此患者未测BCL-6），2例MYC^−^BCL-2^−^（2例患者未测BCL-6）；1例MYC^−^（此例患者未测BCL-2和BCL-6）（图1、表1）。

**图1:**
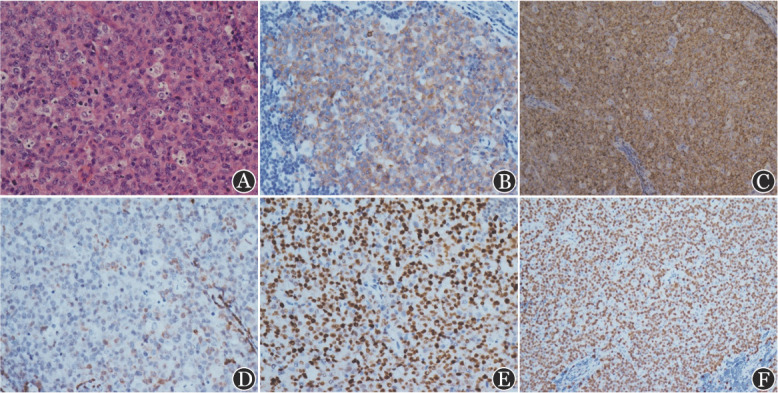
例10右颈部淋巴结病理HE染色（A）及免疫组化染色（B～F）结果 A 淋巴结结构破坏，淋巴样细胞弥漫增生，瘤细胞圆形，体积中等偏大，中等量胞质，细胞核圆形，可见小核仁，核分裂象易见，并见明显凋亡，部分区域可见星空现象（×400）；B CD10（+）（×400）；C CD20（+++）（×200）；D Bcl-2（+/−）（×400）；E Bcl-6（+）（×400）；F Ki-67（90％+）（×200）

**表1:** 11例高级别B细胞淋巴瘤患者的免疫组化和FISH结果

例号	免疫组化								FISH		
	CD10	CD20	Bcl-2	Bcl-6	Ki-67	C-myc	MUM1	TdT	MYC	BCL2	BCL6
1	+	+++	80％+	+	100％+	40％+	+	/	−	−	−
2	+	+++	>90％+	+	90％+	/	/	/	−	−	−
3	+	+	+	+	>90％+	40％+	+	/	−	−	−
4	+	+	弥漫+	+	90％+	10％+	15％+	−	−	−	−
5	+	+	弥漫+	+	100％+	90％+	部分+	−	+	−	−
6	+	+/−	+	+	85％+	/	+	−	−	−	/
7	+	+	−	+	90％+	30％+	/	−	−	−	−
8	−	+	+	弱+	90％+	70％+	+	−	−	−	−
9	+++	+	++	−	>75％+	/	/	/	+	−	/
10	+	+++	+/−	+	90％+	/	/	−	−	−	/
11	+	+	+	+	95％+	/	/	−	−	/	/

注 +：阳性；−：阴性；/：未做

4. 治疗及疗效评估：11例患者中4例患者采用改良NHL-BFM-90方案治疗，6例患者采用改良NHL-BFM-95方案治疗，1例采用R-CODOX-M/R-IVAC方案。

11例患者中10例患者获得完全缓解（CR），1例接受改良NHL-BFM-95方案治疗的患者疗效疾病进展，该例患者尝试多种化疗方案［包括RICE方案（利妥昔单抗、异环磷酰胺、卡铂、依托泊苷）、FAB/LMB方案（长春新碱、环磷酰胺、泼尼松、柔红霉素、甲氨蝶呤、阿糖胞苷、依托泊苷、利妥昔单抗）］联合伊布替尼治疗均无效，最终死于肿瘤进展（存活5.2个月）。中位随访32.5（5.2～139.4）个月，3年OS率和EFS率均为（90.9±8.7）％。

## 讨论

HGBL是一类高侵袭性B细胞淋巴瘤，无特异性临床表现，病理是诊断的金标准，但其形态学复杂，较难辨别时需要结合免疫组化和FISH检测等[7]。按照2016年版WHO的HGBL新分类，本研究中11例均为HGBL，NOS。文献报道该型多见于老年人，恶性程度高，且发病率与年龄呈正相关，男女比例各占50％，高强度化疗可改善预后，但预后差[4]。本研究结果显示该类型也可见于儿童群体，男性多见。有文献报道HGBL伴MYC、BCL-2和（或）BCL-6重排在儿童群体中罕见[4],[8]，本研究的11例患者中无双打击或三打击情况，与文献报道一致。

HGBL在形态学上通常具有中等大小或类胚样细胞，细胞核呈圆形，染色质分散，核不明显或呈BL样。患者的IHC均表达CD20，大多数表达Bcl-6，而CD10、Ki-67、C-myc的表达呈现多样性，大多数患者不表达IRF4及MUM1 [4]。本研究中所有患者均表达CD20，10例患者表达Bcl-6，与文献报道一致。不同的是，本研究所有儿童HGBL患者均表达MUM1，CD10阳性率高（10/11），所有患者Ki-67均较高，且大多数（10/11）患者表达Bcl-2。由于病例数较少，相关文献报道也少，尚不能得出明确结论。

形态学及免疫组化诊断困难时，可以借助FISH用于鉴别诊断。文献报道此类型20％～35％存在MYC重排，伴或不伴MYC拷贝数增加[4]，并且可能携带孤立的BCL-2或BCL-6重排或同时合并BCL-2和BCL-6基因易位[9]。本研究中2例MYC基因重排阳性患者不合并BCL-2或BCL-6基因易位，与既往成人文献报道不一致[4]。可能原因为儿童与成人HGBL的生物学特点和发病机制不同，也可能是病例数较少，需要前瞻性研究数据进一步分析。

HGBL，NOS的研究较少，其临床特点及危险度特征均无定论。本研究结果显示，儿童HGBL好发于腹、盆腔，起病时近半数患儿伴随LDH升高，多数患者为低中危组，提示儿童HGBL易发生结外受累，但肿瘤负荷相对较低。

在治疗和预后方面，本研究中多数患者采用改良的NHL-BFM95方案，总体疗效较好，11例患者中10例疗效达CR，10例患者长期存活，仅1例患者死亡，提示采用根据危险度分层的高强度化疗方案治疗儿童HGBL疗效好，生存率高。而一项回顾性多中心研究显示，成人HGBL的预后差，生存率低于50％[10]。另外，有研究报道，在成人患者中，同时存在MYC及Bcl-2基因异常的患者较单基因异常患者预后差[3]。本研究结果显示，儿童HGBL的预后较成人好，可能与HGBL的生物学特点、基因状态、治疗强度有关。

综上所述，儿童HGBL易发生结外受累，多发生于腹、盆腔，侵袭性强，病理情况复杂，按危险度分层治疗后预后较好，需进一步扩大样本量探讨儿童HGBL的临床特点。
